# Facial electromyography and subjective liking data from 70 New Zealand participants in response to food images and chocolate samples

**DOI:** 10.1016/j.dib.2020.105124

**Published:** 2020-01-11

**Authors:** Elizabeth C. Nath, Peter R. Cannon, Michael C. Philipp

**Affiliations:** School of Psychology, Massey University, Private Bag 11-222, Palmerston North, 4442, New Zealand

**Keywords:** Facial electromyography, Food preference, Food liking, Social context, Implicit emotion

## Abstract

This article describes a dataset of facial electromyography and subjective liking data from 70 New Zealand participants used in the study “An unfamiliar presence reduces facial disgust responses to food stimuli” by Nath, Cannon and Philipp [1]. Participants’ facial muscle activity from zygomaticus major, corrugator supercilii, and levator labii superioris was recorded as they viewed and rated food images, and tasted samples of chocolate. Half of the participants were seated alone, and the other half was seated in the presence of the researcher. The data allows for investigations into the effect of social context on hedonic ratings and facial responses to food, and an exploration into the individual factors contributing to differences in facial reactivity. The data includes raw EMG files generated by Acqknowledge 4.2, raw subjective liking files generated by PsychoPy, a table of participant information, the food images stimuli, the PsychoPy code used for stimuli presentation, and the R scripts used to filter, aggregate and analyse the data.

**Table:** Specifications Table

Subject	Experimental and Cognitive Psychology
Specific subject area	Psychophysiology, consumer psychology
Type of data	Table of participant demographics (.csv file) Food Images (.jpeg files) PsychoPy stimuli presentation program code (.py file) EMG raw data (.acq files) EMG raw data (.csv files) Subjective liking raw data (.csv files) R data processing and analysis code (.R files)
How data were acquired	Facial electromyography data was acquired via a BIOPAC MP150 physiological recording device (BIOPAC Systems Inc., Goleta, CA). Subjective liking data was acquired via a labelled affective magnitude scale [2] presented using a custom PsychoPy program [3].
Data format	Raw data
Parameters for data collection	Data was collected from participants living in Auckland, NZ, aged 18 and above, and who were fluent in English.
Description of data collection	Participants' facial muscle activity from zygomaticus major, corrugator supercilii and levator labii superioris were recorded while viewing thirty food images and consuming two small pieces of chocolate either alone, or in the presence of the researcher.
Data source location	Institution: Massey University City/Town/Region: Albany, Auckland Country: New Zealand Latitude and longitude (and GPS coordinates) for collected samples/data:] −36.73572, 174.6922
Data accessibility	Repository name: Zenodo Data identification number: 1208749 Direct URL to data: https://zenodo.org/record/3462883
Related research article	Elizabeth C. Nath, Peter R. Cannon, Michael C. Philipp An unfamiliar social presence reduces facial disgust responses to food stimuli Food Research International https://doi.org/10.1016/j.foodres.2019.108662

**Table:** 

**Value of the Data** • The data consists of recordings from three facial muscles involved in emotional facial expressions and other functional facial movement. It allows for a richer understanding of how food stimuli, social context and individual differences influence facial expression. • Emotion researchers can use this data to better understand individual differences in facial expression generation. Consumer researchers can use this data to better understand hedonic and implicit emotion responses to food stimuli. • These data allows for investigations into the effect of social context on hedonic ratings and facial responses to food, and the demographic information allows for explorations into the individual factors contributing to differences in facial reactivity. • Muscle activity data during the consumption of chocolate samples allows for investigations into the effect of sensory properties of chocolate such as hardness, bitterness, fat content and melting point on functional and emotional facial muscle activity.

## 1 Data

The dataset provided with this article contains raw facial electromyography data from the zygomaticus major, corrugator supercilii and levator labii superioris muscles of 70 participants as they viewed 30 food images and sampled two pieces of chocolate [1]. Electromyography data is provided in both.acq and.csv file formats. Participant information and their assigned experimental condition is included in a separate.csv file. The order of presentation of the food images was randomized for each participant and this order is contained in each participants’.csv file. The food image stimuli are included as.jpeg files. The experiment instructions and stimuli were presented via PsychoPy using a custom program; this code is included as a.py file. The R scripts used to filter, aggregate, and analyse the data are included as. R files. Given that this data was collected in New Zealand, where the mains electricity frequency is 50 Hz, it will require band stop filters every 50Hz to remove mains frequency interference and harmonics prior to analysis.

In the marker channel of each .acq file, each unique marker corresponds to a trial event period as in Table 1 below.

**Table 1:** Marker number allocations for .acq files.

Marker number	Trial event
10	Food image baseline
20	Food image
30	Dark chocolate consumption
40	Milk chocolate consumption
100	Corrugator supercilii maximum voluntary contractions
150	Levator labii superioris maximum voluntary contractions
200	Zygomaticus major maximum voluntary contractions

## 2 Experimental design, materials, and methods

### 2.1 Participants

Seventy participants (52 women, 18 men) aged between 18 and 74 (M = 28.8, SD = 10.0) and primarily of Asian and NZ European descent were recruited via advertisements placed around the university campus and on local community Facebook groups. Advertisements were in English and asked for participants to be 18 years and older. No other exclusion criteria were stipulated. All participants gave written consent and were compensated for their time with a NZ$15 department store or supermarket gift card.

### 2.2 Design

**Fig. 1:**
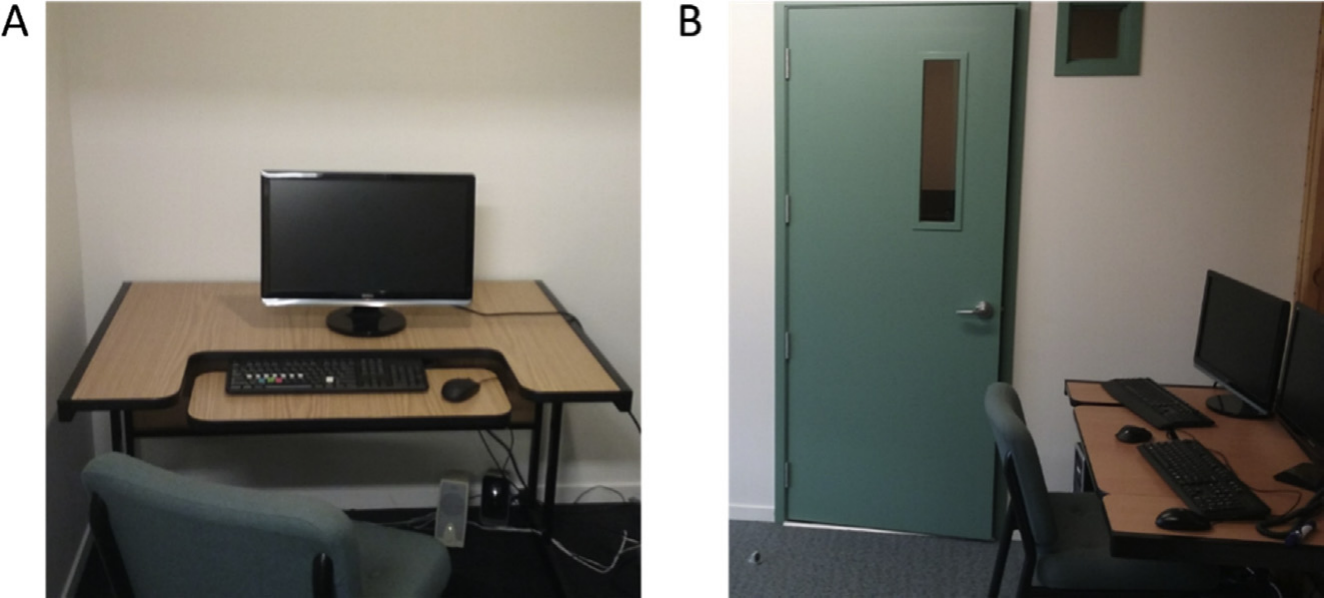
(A) Experimental setup for participants in the Alone condition. (B) Experimental setup for participants in the Observed condition. The green door leads into the testing booth depicted in photograph A.

Participants were randomly assigned to one of two experimental conditions – alone or observed. Participants in the alone condition were directed to sit in front of a computer screen in a sound-attenuated testing booth (Fig. 1A) while participants in the observed condition were directed to sit in front of a computer screen beside the experimenter in the main lab (Fig. 1B).

### 2.3 Stimuli

Thirty food images were selected such that their perceived acceptability would likely be distributed along the entire valence scale (ranging from greatest imaginable like to greatest imaginable dislike). In their study on food images and acceptability conducted in Auckland, New Zealand, Li [4] found that the local sample rated images of familiar foods such as cake, roast chicken and chocolate highest in acceptability and rated images of unfamiliar foods from foreign cultures such as Balut (Philippines) and Svið (Iceland) lowest in acceptability. The images were presented with PsychoPy [3] on a 60.5 cm Philips monitor with 1920 × 1080 resolution and a 60 Hz frame rate. The chocolate stimuli consisted of one 3g piece of Whittaker's 62% Dark Cocoa Block, followed by one 3g piece of Whittaker's 33% Creamy Milk Block. The order of presentation of the chocolate stimuli was always in this sequence (i.e. not randomised for each participant). The timing of trial events was synchronised with the psychophysiological recording software via parallel port. All images used in the present study are licensed for reuse.

### 2.4 Measures

#### 2.4.1 Subjective liking ratings

Subjective liking was measured using the labelled affective magnitude scale (LAM), developed by Schutz and Cardello [2] to measure food liking. The LAM is a vertical, 100-point, line scale with 11 semantic anchors ranging from “greatest imaginable dislike” at 0, to “greatest imaginable like” at 100.

#### 2.4.2 Facial electromyography

**Fig. 2:**
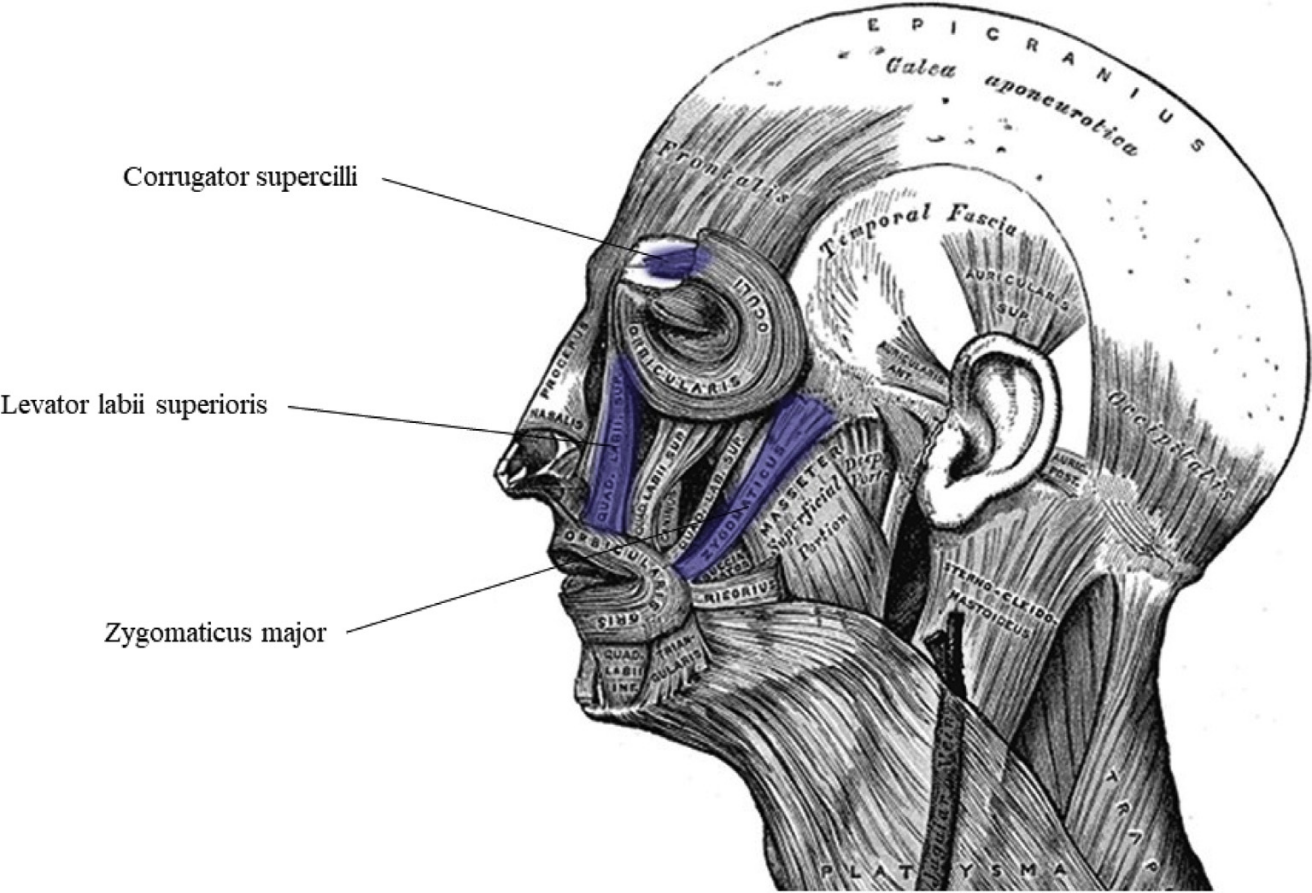
Position of corrugator supercilli, levatorlabi superioris and zygomaticus major muscles. Adapted from Anatomy of the Human Body by H. Grey, 1918, Philadelphia, PA: Lea &Febiger.

Muscle activity from zygomaticus major (contraction lifts corner of mouth during smiling), corrugator supercilii (contraction knits brow during frowning) and levator labii superioris (contraction wrinkles nose during expressions of disgust) [5] on the left side of the face was recorded using a BIOPAC MP150 physiological recording device, three BIOPAC amplifiers, and six 4 mm Ag/AgCl reusable surface electrodes (BIOPAC Systems Inc., Goleta, CA). The approximate location of each pair of electrodes is indicated in Fig. 2. An 8 mm ground electrode was attached to the forehead near the hairline.

### 2.5 Procedure

**Fig. 3:**
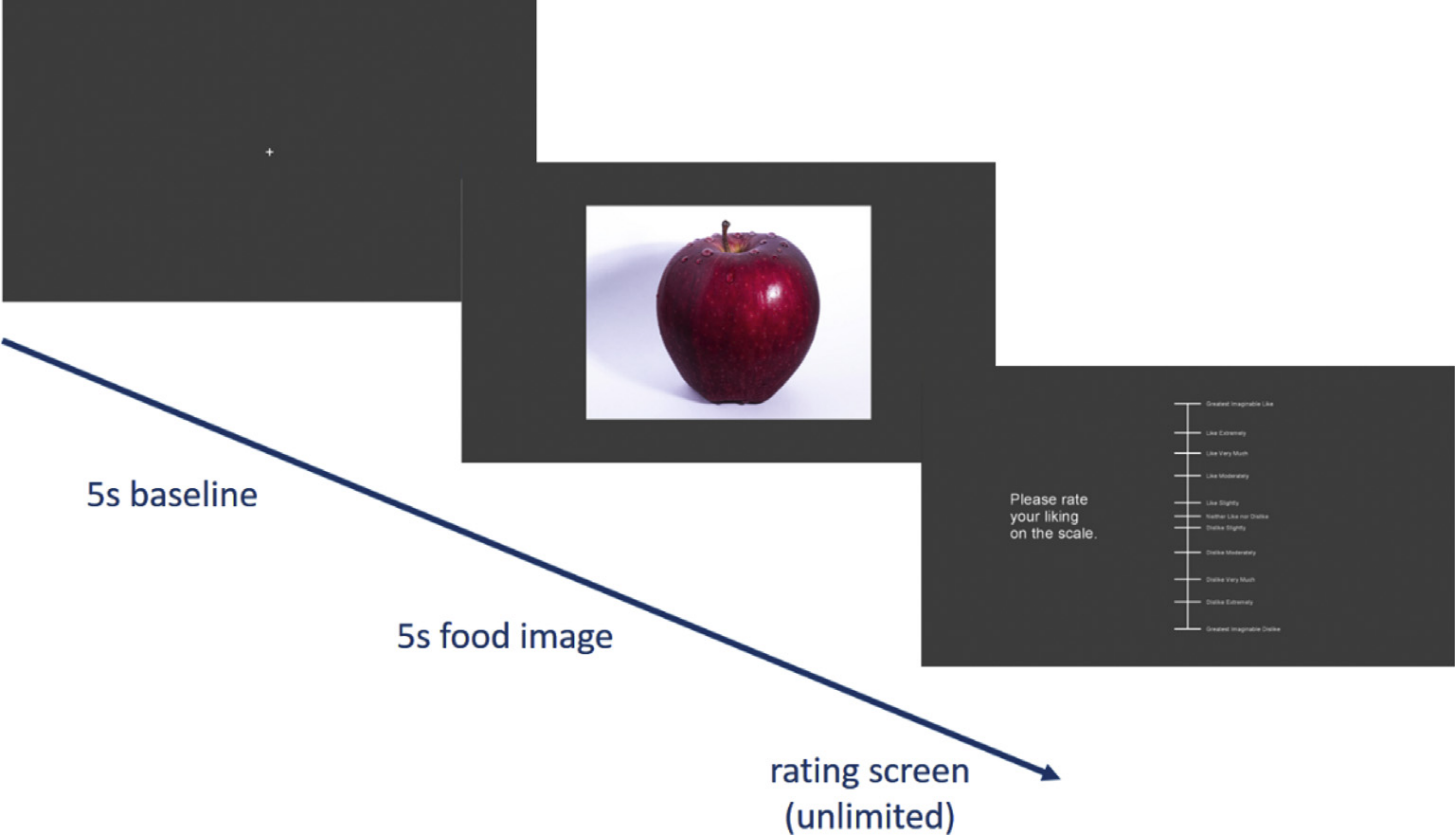
Sequence of trial events. Experimental stimuli was programmed and presented in PsychoPy [3].

Upon expressing interest in the study, potential participants were emailed a study information sheet describing the experimental procedure. The information sheet explained that the purpose of the study was to investigate facial behaviours in response to food images and as such, participants were blind to the social context manipulation. On arrival at the lab, each participant was asked to sign a consent form and given a NZD$15 gift card. To prepare the skin sites for electrode adhesion, participants were first asked to wash their face with a mild facial cleanser. Then, the skin sites were cleaned with an alcohol swab and abraded with an abrading pad. A small amount of electrode gel was applied to each skin site with a cotton bud and any excess gel was wiped off with a tissue. Once the electrodes were attached, participants in the alone condition were directed to sit in front of a computer screen in a sound-attenuated testing booth while participants in the observed condition were directed to sit in front of a computer screen beside the experimenter in the main lab. The participants' chair was positioned such that their faces were between 60 and 70 cm from the monitor. In the observed condition, the experimenter sat silently beside the participant facing the screen and maintained a neutral expression and body language. The seating arrangement was such that direct eye contact between the participant and experimenter was not possible. In both conditions, the experimenter ran a simulation of the experiment and demonstrated response requirements before starting the main experiment. In the main experiment, participants viewed thirty food images presented in a randomised order and rated their liking of each food image on the LAM scale. Each trial consisted of a fixation screen displayed for 5000 ms (a white “+” presented in the centre of the screen against a grey background), a food image displayed for 5000 ms (a 1024 × 768 pixel image presented in the centre of the screen against a grey background) and a rating screen (the LAM scale in white font against a grey background) displayed until the participant made a response (refer to Fig. 3 for an illustration). Participants were required to indicate their liking with a single mouse click on the scale. Following the food images, participants were asked to sample a piece of dark chocolate and a piece of milk chocolate (approximately 3 g in weight) and rate their liking on the LAM scale. After participants rated the food images and chocolate samples, maximum facial muscle contractions were recorded with the aid of on-screen prompts to pose a frown, wrinkle the nose and smile. Finally, the experimenter removed the electrodes and debriefed participants, explaining the full objectives of the study.

### 2.6 Data processing

The EMG signals were relayed through shielded cable to Biopac amplifiers set to a gain of 5000 with a high pass filter at 1 Hz. The signal was digitally recorded at 2000 Hz using Acqknowledge 4.2 software. Raw data files were uploaded regularly during the data collection phase to a Zenodo online depository (https://zenodo.org/record/3462883) and are available under the Creative Commons Attribution Share-Alike 4.0 licence.
